# The ICP27 Homology Domain of the Human Cytomegalovirus Protein UL69 Adopts a Dimer-of-Dimers Structure

**DOI:** 10.1128/mBio.01112-18

**Published:** 2018-06-19

**Authors:** Richard B. Tunnicliffe, Richard F. Collins, Hilda D. Ruiz Nivia, Rozanne M. Sandri-Goldin, Alexander P. Golovanov

**Affiliations:** a Manchester Institute of Biotechnology, The University of Manchester, Manchester, United Kingdom; b School of Chemistry, Faculty of Science and Engineering, The University of Manchester, Manchester, United Kingdom; c Electron Microscopy Facility, Faculty of Biology, Medicine and Health, The University of Manchester, Manchester, United Kingdom; d Biomolecular Analysis Core Facility, Faculty of Biology, Medicine and Health, The University of Manchester, Manchester, United Kingdom; e Department of Microbiology and Molecular Genetics, School of Medicine, University of California, Irvine, California, USA; Princeton University

**Keywords:** human cytomegalovirus, ICP27 homology domain, structural analysis, tetramerization, transmission electron microscopy

## Abstract

The UL69 protein from human cytomegalovirus (HCMV) is a multifunctional regulatory protein and a member of the ICP27 protein family conserved throughout herpesviruses. UL69 plays many roles during productive infection, including the regulation of viral gene expression, nuclear export of intronless viral RNAs, and control of host cell cycle progression. Throughout the ICP27 protein family, an ability to self-associate is correlated with the functions of these proteins in transactivating certain viral genes. Here, we determined the domain boundaries of a globular ICP27 homology domain of UL69, which mediates self-association, and characterized the oligomeric state of the isolated domain. Size exclusion chromatography coupled with multiangle light scattering (SEC-MALS) revealed that residues 200 to 540 form a stable homo-tetramer, whereas a shorter region comprising residues 248 to 536 forms a homo-dimer. Structural analysis of the UL69 tetramer by transmission electron microscopy (TEM) revealed a dimer-of-dimers three-dimensional envelope with bridge features likely from a region of the protein unique to betaherpesviruses. The data provide a structural template for tetramerization and improve our understanding of the structural diversity and features necessary for self-association within UL69 and the ICP27 family.

## IMPORTANCE

Human cytomegalovirus (HCMV) infection is widespread in the human population but typically remains dormant in an asymptomatic latent state. HCMV causes disease in neonates and adults with suppressed or impaired immune function, as the virus is activated into a lytic state. All species of herpesvirus express a protein from the ICP27 family which functions as a posttranscriptional activator in the lytic state. In HCMV, this protein is called UL69. The region of sequence conservation in the ICP27 family is a folded domain that mediates protein interactions, including self-association and functions in transactivation. All members thus far analyzed homo-dimerize, with the exception of UL69, which forms higher-order oligomers. Here, we use biochemical and structural data to reveal that UL69 forms stable tetramers composed of a dimer of dimers and determine a region essential for cross-dimer stabilization.

## OBSERVATION

Human cytomegalovirus (HCMV) causes a variety of diseases in newborns infected *in utero* and in immunocompromised individuals, such as HIV/AIDS patients and recipients of transplants. In common with all herpesviruses, HCMV causes lifelong infections and has high prevalence. Early in infection, HCMV expresses a multifunctional regulatory protein, UL69, which is the prototype mRNA export factor from the betaherpesvirus subfamily. UL69 is a member of the ICP27 family (named after the prototype from herpes simplex virus [HSV]), a group of functionally similar proteins that have homologues in all herpesviruses that have been sequenced (1, 2). UL69 is present within the viral tegument and is released directly into the host cell upon infection (2–4). UL69 has multiple functions in viral gene transcription and translation regulation, and it also influences cell cycle progression (5). While the mechanistic details are not fully understood, studies have identified several functionally important UL69 interaction partners. One binding partner is the cellular transcriptional elongation factor SPT6, which functions in chromatin remodeling, transcript elongation, and mRNA export (6). UL69 also binds the cellular helicase UAP56, which functions in transcription elongation and mRNA export, and the interaction increases the efficiency of mRNA export (7–9). UL69 also binds protein-arginine methyltransferase 6 (PRMT6) on the same site as UAP56 (10). UL69 contains arginine-rich motifs which directly mediate RNA interactions, although this interaction is not crucial for the nuclear export of transcripts, as mutants that do not bind RNA are still able to efficiently export unspliced viral mRNA (11). It also has nuclear export and nuclear localization sequences that facilitate shuttling between the nucleus and cytoplasm (12). The localization of UL69 is further modulated by posttranslation modification by cellular cyclin-dependent kinases and the HCMV orthologue kinase UL97 (13–17). Additionally, UL69 interacts with eIF4A1, part of the cap-binding complex, and poly(A) binding protein to promote translation (18). Thus, UL69 forms numerous functionally important interactions with cellular proteins, allowing it to act posttranscriptionally to mediate the nuclear export and cytoplasmic accumulation of viral mRNAs and also promote their translation.

HCMV UL69 is composed of 744 amino acids (aa) and contains a homologous folded region termed the ICP27 homology domain (IHD). The IHD is located centrally in UL69 and is flanked by predicted intrinsically disordered regions, whereas in alpha- and gammaherpesvirus homologues, the IHD is at the C terminus, with one disordered region in the N terminus. The IHD mediates protein interactions, such as UL69 binding to SPT6, and also self-association. The IHD in HSV-1, varicella zoster virus (VZV), Epstein-Barr virus (EBV), and Kaposi’s sarcoma-associated herpesvirus (KSHV) homologues also mediates self-association (19–23). Self-association is functionally important, as mutants of ICP27 and its homologues that are unable to homo-oligomerize are defective in their transactivation functions (20, 22, 24) and protein interactions, such as those of SR proteins with ICP27 (25). UL69 self-association has been observed in yeast two-hybrid screens, and truncations suggested that the domain responsible was contained within residues 269 to 574, precisely the region that interacts with SPT6, suggestive of a correlation between self-association and this protein interaction (6). Analytic size exclusion chromatography (SEC) of full-length UL69 suggested that tetramers or high-order oligomers were present (21). In contrast, the oligomerization state of homologous herpesvirus proteins appears quite different; analytic gel filtration experiments suggested that the isolated KSHV open reading frame 57 (ORF57) IHD forms only homo-dimers (26). The isolated IHD from HSV-1 ICP27 has been analyzed by SEC coupled with multiangle light scattering (MALS) and analytical ultracentrifugation (AUC) experiments by two independent groups, which indicated that it also forms homo-dimers (27, 28). The crystal structures of the IHD of HSV-1 ICP27 revealed a globular symmetrical homo-dimer of approximately 270 residues per protein chain, which adopts a novel protein fold containing a CHCC zinc finger. The dimer interface was extensive and was in part stabilized by domain swap features, including two extended N-terminal α-helices (27, 28). Due to the lack of structural characterization of UL69 and homologues, the reasons for the different oligomerization behavior of the similar IHD from UL69 were not clear. Therefore, to inform future studies to probe the functional importance of this structural feature, we performed biochemical and structural analysis revealing regions necessary for tetramer formation.

### Characterization of UL69 self-association.

**FIG 1:**
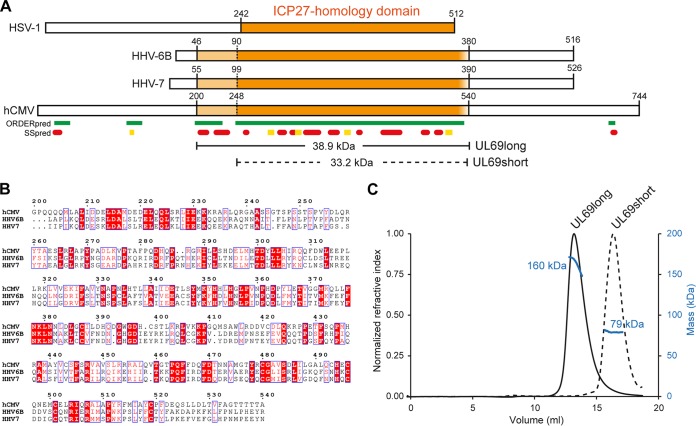
HCMV UL69 self-associates via a conserved ICP27 homology domain and forms a tetramer. (A) Location of the ICP27 homology domain (IHD) (orange) with the prototype from HSV-1 and the homologues from HHV-6A, HHV-6B, and HCMV. Prediction within HCMV UL69 of ordered/structured regions is indicated by green blocks, and the secondary structure is shown by red cylinders for α-helices and yellow blocks for β-sheets (29). The domain boundaries for constructs used here are indicated along with the molecular weight based on the primary sequence (30). (B) Primary sequence alignment of IHDs from UL69 homologues within the betaherpesvirus subfamily, produced with ESPrint (35). (c) SEC-MALS analysis of the molecular weights of purified UL69 constructs indicates that tetramer formation within UL69long is destabilized to a dimer by truncation of the region from aa 200 to 247 in UL69short.

To confirm and characterize the oligomeric state of UL69 mediated by the IHD, first we established the likely domain boundaries *in silico* using sequence comparisons combined with predictions of disorder and structure using Clustal Omega and PSIPRED, respectively (29, 30). The results suggested that two regions may contain the IHD; residues 200 to 540 or a shorter segment of residues 252 to 536 (Fig. 1A and B). We cloned constructs encoding UL69 residues 200 to 540 and UL69 residues 248 to 536 (named UL69long and UL69short, respectively); the former contained the IHD along with an N-terminal region conserved only in betaherpesviruses, whereas the latter short construct contained only the IHD. The proteins were expressed and purified to homogeneity and analyzed by SEC-MALS. The data indicated that the UL69long form was 160 kDa, indicative of a homo-tetramer, and that UL69short was 79 kDa, likely a homo-dimer (Fig. 1). The mass of UL69short is above the predicted mass for a pure homo-dimer (66.4 kDa), suggestive of some residual coelution with a higher-order UL69 oligomer, possibly a residual ability to form a transient tetramer. Thus, UL69 can form a tetramer, and the region from aa 200 to 247, which is not in the shorter construct, is required for stable tetramer formation. This suggested that the tetramer may be formed by the association of two dimers.

### TEM analysis of the UL69 ICP27 homology domain.

**FIG 2:**
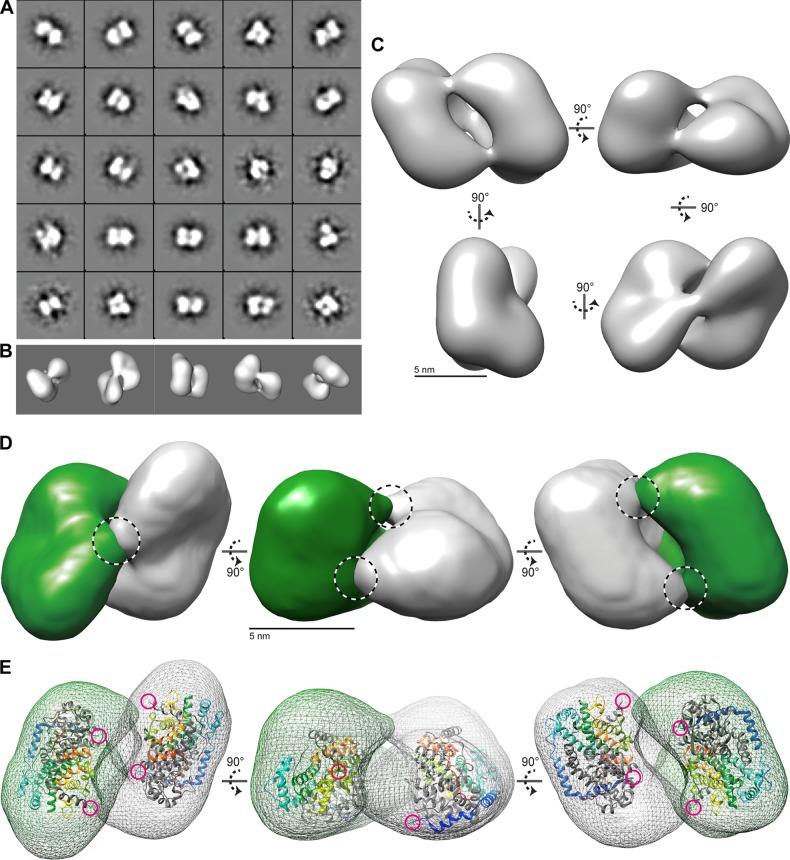
3D structure of the UL69 tetramer determined by 3D reconstruction of negative-stain EM images. (A) A selection of 25 projection-class averages were calculated using reference-free multivariate statistical analysis (MSA). Box size = 33 nm. (B) Surface-rendered views of the final 3D reconstruction of the UL69long envelope orientated to match the bottom row initial class averages of panel A. (C) Surface-rendered views of the UL69 long structure. Five-nanometer scale bars are shown. (D) Segregation of the UL69long structure into two lobe volumes related by C_2_ symmetry and colored green and gray. Proposed bridge features are highlighted by dashed circles. (E) Each lobe of UL69 was fitted to the ICP27 IHD dimer coordinates. The surface of UL69long is shown as a mesh, and ICP27 (PDB accession number 4YXP) is shown as a ribbon trace, with one chain of the homo-dimer colored gray and the second chain in rainbow colors (blue to red, from the N terminus to the C terminus). The N termini of ICP27 are further highlighted by magenta circles, highlighting their proximity in space to each other and to the bridge regions.

To explore the structure of the UL69long 160-kDa tetramer, we used single-particle transmission electron microscopy (TEM). Negatively stained UL69long particles (see Fig. S1 and S2 in the supplemental material) were classified using reference-free alignment and used to calculate a three-dimensional (3D) reconstruction (Fig. 2). The structure was visualized using UCSF Chimera, which allowed us to examine the UL69 molecular envelope and overall protein distribution of a C_2_ symmetrical oligomer of approximately 130 Å by 90 Å by 70 Å. The complex is composed of two major volumes, each appearing as a lobe the shape of a kidney bean, with the concave faces at the interior contact surface (31) (Fig. 2C). We did consider the likelihood that the complex had D_2_ symmetry, with each dimer having an additional axis of rotational symmetry; however, at the resolution of the study, unambiguous assignment was not possible, and therefore C_2_ symmetry was conservatively applied (Fig. S3). The UL69long 3D envelope could readily be assigned to the two lobe volumes using the Chimera segment tool, and each lobe was fitted with an ICP27 IHD dimer using Chimera (32) (Fig. 2D and E). Some additional peripheral volume was not occupied by the 60-kDa ICP27 IHD dimer, which was consistent with the higher molecular weight of the UL69long dimer construct used (78 kDa). The data therefore correlated with a tetramer composed of a dimer of dimers. The dimers are connected by three narrow bridges suggestive of dimer-dimer contacts; the ICP27 N-terminal helices point toward two of the bridges. ICP27, which forms dimers, lacks the region equivalent to residues 200 to 247 of UL69long; removal of this region in the UL69short construct prevents tetramer formation for UL69 as well (Fig. 1C). We hypothesize that the bridge features may in part be formed by N-terminal residues 200 to 247, and additional dimer-dimer contacts between the two core globular domains likely also occur.

10.1128/mBio.01112-18.1 FIG S1  TEM negative-stain field, with individual UL69long oligomer particles indicated by white boxes. Download FIG S1, TIF file, 1.1 MB. Copyright © 2018 Tunnicliffe et al. 2018 Tunnicliffe et al. This content is distributed under the terms of the Creative Commons Attribution 4.0 International license.

10.1128/mBio.01112-18.2 FIG S2  Sample data of raw individual boxed UL69 complex particles. Download FIG S2, TIF file, 1 MB. Copyright © 2018 Tunnicliffe et al. 2018 Tunnicliffe et al. This content is distributed under the terms of the Creative Commons Attribution 4.0 International license.

10.1128/mBio.01112-18.3 FIG S3  Orthogonal views of the UL69long envelope obtained by TEM illustrating the C_2_ rotational symmetry present in the structure. Hypothetical D_2_ dihedral symmetry as indicated was not evident in the data and therefore could not be justified. Download FIG S3, TIF file, 1.5 MB. Copyright © 2018 Tunnicliffe et al. 2018 Tunnicliffe et al. This content is distributed under the terms of the Creative Commons Attribution 4.0 International license.

### Conclusions.

The SEC-MALS and TEM data indicate that the UL69 IHD forms a tetramer composed of a dimer of dimers, further corroborating previous estimates of the UL69 oligomeric state by Lischka et al. (21). The bridge region from aa 200 to 247 is necessary for stable tetramer formation but is not part of the core homo-dimer IHD. Secondary-structure predictions suggest that the bridge region is α-helical and amphipathic in nature and might extend and contact an adjacent dimer, facilitating stable tetramer formation. Interestingly, primary sequence analysis using the LOGICOIL program predicted that residues 202 to 244 adopt a coiled-coil structure, a feature that contributes to oligomerization (33). The bridge region is conserved within homologues in betaherpesviruses, and therefore the tetramer structure observed here is likely a feature of the subfamily (Fig. 1B). The functional importance of the ability of HCMV UL69 and other ICP27 homologues from human herpesvirus 6A/B (HHV-6A/B) and HHV-7 to form homo-tetramers is currently unknown. Therefore, we hope that the data inform future studies that may probe tetramer formation through deletion of the bridge region or point mutation of conserved hydrophobic residues that likely mediate cross-dimer stabilization. One can speculate that higher-order oligomers may allow increased synergy or complexity within UL69 interactions, as the protein might feasibly switch between dimer and tetramer, thus presenting different binding surfaces and broadening the range of interactions possible with other proteins and nucleic acids relative to the IHDs from alpha- and gammaherpesviruses, which form only homo-dimers.

### Methods. (i) Protein expression and purification.

Two DNA fragments encoding HCMV UL69 residues 200 to 540 and UL69 residues 248 to 536 were obtained by gene synthesis (Invitrogen). Both constructs also contained an HRV3C protease-cleavable N-terminal thioredoxin tag plus Strep-tag and were codon optimized for expression in Escherichia coli. Constructs were cloned into the pET-21a expression vector and purified by Strep-tag affinity, and then the thioredoxin tag was cleaved as described previously for ICP27 (28). Protein was further purified by gel filtration using a Superdex 200 26/600 column (GE Healthcare) preequilibrated in gel filtration buffer [20 mM HEPES, 150 mM NaCl, 50 mM l-Arg, 50 mM l-Glu 49, 1 mM Tris(2-carboxyethyl)phosphine hydrochloride (TCEP), pH 7.9].

### (ii) SEC-MALS.

Purified UL69long and UL69short were analyzed by SEC-MALS; samples (0.5 ml at 1 mg/ml) were loaded onto a Superdex 200 10/300 GL column (GE Life Sciences; 0.75 ml/min in gel filtration buffer) and passed through an Effective Optical Systems (EOS), Wyatt Technology Dawn Heleos II 18-angle laser photometer coupled to a Wyatt Optilab rEX refractive index detector. Data were analyzed using Astra 6 software (Wyatt Technology Corp., CA).

### (iii) TEM.

To explore the structure of the UL69long 160-kDa tetramer, a purified sample was dialyzed into HEPES buffer (20 mM HEPES, 150 mM NaCl, pH 7.4), and then the protein (10 µg/ml) was adsorbed onto glow-discharged carbon-coated grids and stained with 4% (wt/vol) uranyl acetate (pH 4.5). Data were recorded at 30,000× on a Tecnai Biotwin instrument at 120 kV with a Gatan Orius charge-coupled device (CCD) camera. Images were recorded with a 1-s exposure at defocus values of 0.5 to 1.6 µm at 2.1 Å per pixel (see Fig. S1 and S2 in the supplemental material). Single-particle analysis was performed using EMAN2 (34). A total of 5,000 particles were selected using semiautomated picking. Following contrast transfer function correction, each data set was subjected to two-dimensional (2D) classification. A total of 25 projection averages were selected and used to generate an initial 3D model (Fig. 2A). This model was used as a start seed for eight rounds of iterative refinement to produce a self-consistent 3D structure with an estimated resolution of 25 to 50 Å, which was consistent with the level of detail observed and the relatively small size of the complex.
