# Supplementary figures and images for: The ICP27 Homology Domain of the Human Cytomegalovirus Protein UL69 Adopts a Dimer-of-Dimers Structure
Source: mBio. 2018 Jun 19;9(3):e01112-18. doi: 10.1128/mBio.01112-18 (PMC6016253; doi:10.1128/mBio.01112-18)

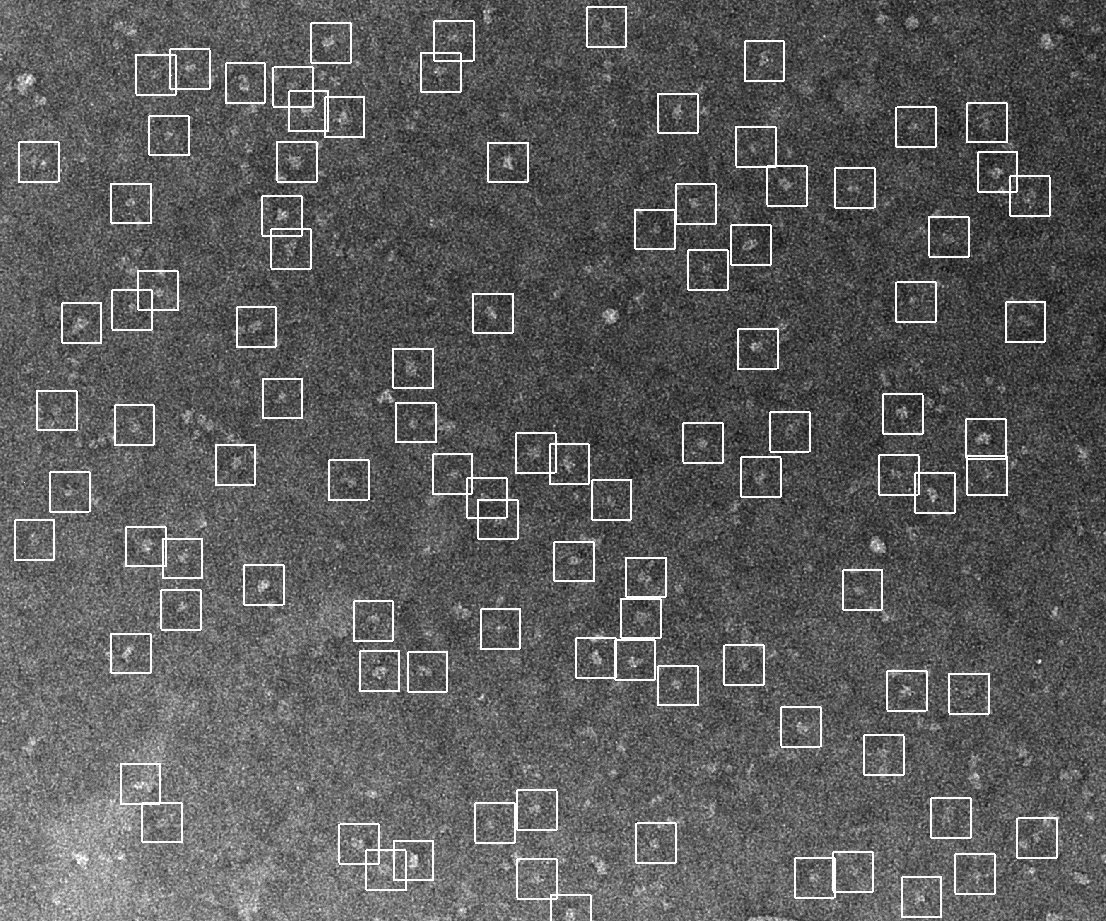

Supplement: FIG S1 [file mbo003183945sf1.tif]

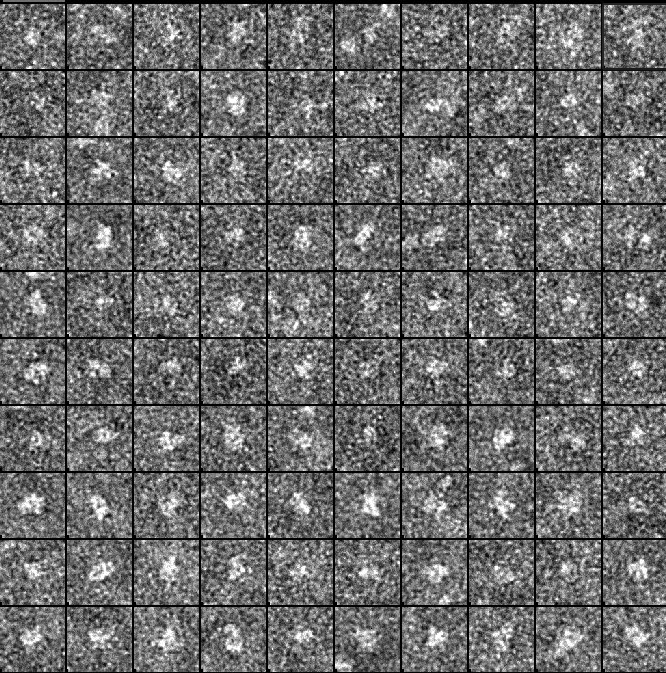

Supplement: FIG S2 [file mbo003183945sf2.tif]

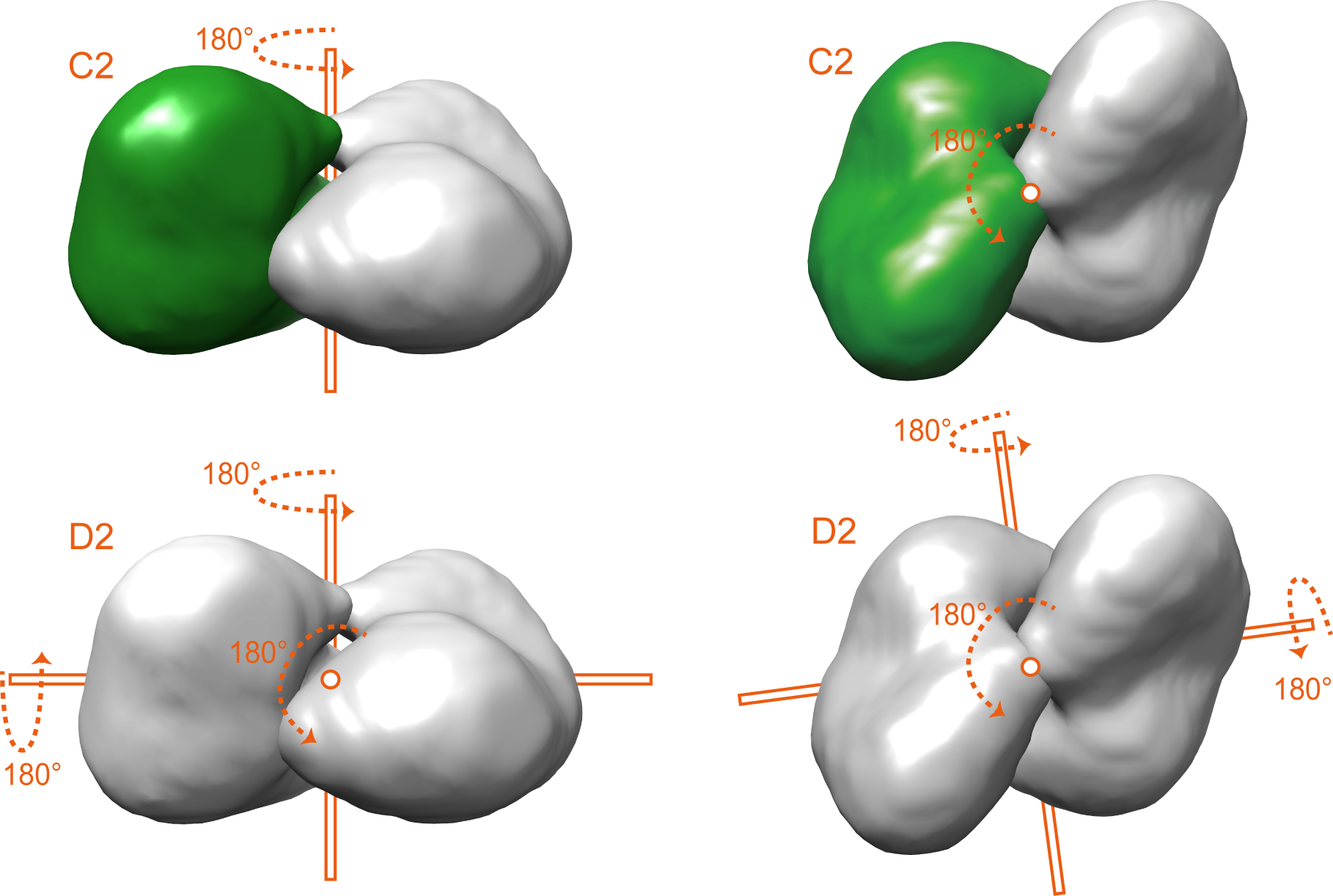

Supplement: FIG S3 [file mbo003183945sf3.tif]
